# Comment on: Modular titanium alloy neck failure in total hip replacement

**DOI:** 10.1051/sicotj/2016032

**Published:** 2017-03-17

**Authors:** Francesco Benazzo, Loris Perticarini

**Affiliations:** 1 Fondazione IRCCS Policlinico San Matteo Pavia Italy

To the Editor of the SICOT-J Orthopaedic Journal,

I am writing to you as I would like to kindly express some considerations on the case-report of Dr. Marco Ceretti on “*Modular titanium alloy neck failure in total hip replacement*” published in your on-line journal last April (M. Ceretti and F. Falez SICOT J 2016, **2**, 20).

The Authors report on “*a case of a repetitive modular femoral neck prosthesis fracture*”; they had initially revised a broken modular Metha stem with a MODULUS stem, neck 135° long, head 36 medium in a 43-year-old woman with body mass index (BMI) 38.6 kg/m^2^ (weight 110 kg, height 170 cm); both stems presented modular necks in titanium alloy. The MODULUS stem was then revised with a Wagner monoblock uncemented titanium stem following the fracture at the neck-stem junction.

This article reports a clinical case but it does not include any laboratory evidence in terms of fracture analysis to support the Authors’ conclusions and to elucidate the mechanisms of failure. No retrieval analysis on the two stems seems to have been in fact carried out, such as standard macroscopic inspection and observations, standard corrosion testing, micro analysis of the implant alloy, light microscopy, Scanning Electron Microscopy (SEM) and patient’s blood sample to check the release of metal ions.

Besides, several publications nowadays report comparative analysis of cobalt-chromium and titanium modular femoral necks, showing that the first material present “*improved resistance against fretting*” with “*lower incidence of surface micromotions*” (e.g. [1, 2]).

Nevertheless, the fracture of these two modular necks should be attributable to an error of indication and not to a failure of the devices themselves, meant to be used in patients with lower BMI.

The surgical technique of the MODULUS stem reports:

“Complications or failures of the total hip replacement may occur in heavy and highly active patients and high offset combinations. The surgeon should perform a careful evaluation of the patient’s clinical condition and level of physical activity before performing hip replacement. Patients who are overweight and/or have high activity levels may not be candidates for hip replacement with modular stems. Alternative devices, such as monoblock hip stems, should be used, when possible, in these patients.”

The available literature sources clearly support these indications on the use of modular stems in the current clinical practice:

“Among patients treated with the titanium alloy neck adapter, a combination of different parameters was identified as risk factors of implant failure. The parameters are intraoperative particle contamination of the cone connection, excessive loading due to a patient weight above 100 kg or high activity level, and male gender. In addition, the risk for failure rises with CCD angles of the cone adapter of 135° and smaller.”

“Contamination with cortical bone particles in the interface was found to be the main influence factor on the fatigue behaviour of the titanium alloy neck adapter.” [1]

“Various circumstances such as large femoral head with a long varus neck, corrosion, patients’ BMI, and activity level may participate in creating the necessary environment for fatigue failure of a modular hip replacement implant.” [3]

Furthermore, I would like to take this opportunity to kindly bring to the attention of your Editorial Office that no failures of the MODULUS stem are recorded in some series of publications concerning clinical experience with this medical device, as indicated in the following articles [4–7].

## Conflict of interest

One of the author is a consultant of Lima Corporate as a Scientific Adviser.
